# iPiG: Integrating Peptide Spectrum Matches into Genome Browser Visualizations

**DOI:** 10.1371/journal.pone.0050246

**Published:** 2012-12-04

**Authors:** Mathias Kuhring, Bernhard Y. Renard

**Affiliations:** Research Group Bioinformatics (NG4), Robert Koch-Institute, Berlin, Germany; University of Utah, United States of America

## Abstract

Proteogenomic approaches have gained increasing popularity, however it is still difficult to integrate mass spectrometry identifications with genomic data due to differing data formats. To address this difficulty, we introduce iPiG as a tool for the integration of peptide identifications from mass spectrometry experiments into existing genome browser visualizations. Thereby, the concurrent analysis of proteomic and genomic data is simplified and proteomic results can directly be compared to genomic data. iPiG is freely available from https://sourceforge.net/projects/ipig/. It is implemented in Java and can be run as a stand-alone tool with a graphical user-interface or integrated into existing workflows. Supplementary data are available at PLOS ONE online.

## Introduction

The field of proteogenomics has gained popularity with the increasing availability of genomic and proteomic data from high-throughput sequencing technologies and tandem mass spectrometry. The joint analysis of proteomic and genomic data allows the direct comparison of expression levels. Common applications are further targeted at gene finding and gene annotation since genes or transcripts can be validated by comprehensive sets of peptides [1], [2].

While the import of genomic data to genome browsers [3]–[8] is facilitated through standardized formats, the import of proteomic data from mass spectrometry measurements remains difficult and currently requires significant manual interaction. A general challenge arises since the exact positions of the origin of a peptide identification in the genome is not necessarily provided by commonly used mass spectrometry data processing software. Thus, significant mapping either based on existing annotation or the underlying sequence is necessary. Further, additional information regarding the identification quality can be of high value in a visualization to assess the reliability.

Kalume et al. [9] use searches against protein databases as well as searches against all six-frame translations from genome databases to confirm and correct existing genes or transcripts by peptide spectrum matches (PSMs). They concentrate on the annotation of a particular organism and make their results available in the Ensembl Genome Browser [4] using the Distributed Annotation System (DAS) [10]. However, they rely on a string search based integration and in-house Python scripts. The Proteogenomic Mapping Tool [11] aims at identifying new genes by applying a string search algorithm on translations of all six reading frames of the desired genome. It does not consider any reference and quality information of peptide spectrum matches and the output is not suitable for a direct visualization in a genome browser.

We developed the ***i*** *ntegrating* ***P*** *eptide spectrum matches* ***i*** *nto* ***G*** *enome browser visualizations* (iPiG) tool to provide an easy-to-use and fast tool with a graphical user interface that facilitates the visualization of peptide identifications in genome browsers. It takes advantage of protein references and enriches the visualization with secondary information.

## Methods

iPiG is designed as an offline tool and all data remains on the client side. The workflow is illustrated in Supplementary S1. Overall, up to five input files are required. A set of identified peptides (PSMs) is imported in either mzIdentML or text format. mzIdentML is the open standard format for peptide identifications [12] and is supported by most identification software or converters [13]. iPiG does not include an integrated postprocessing of peptide identifications, but relies on prior false-discovery rate estimation procedures [14], [15] and incorporates their results in the visualization. An UniProt id mapping file that entails the matching of protein and gene identifiers can specify the peptide-gene linkage and can further be supported by additional information from the headers in a fasta file (e.g. gene names/symbols). iPiG can also be applied without these files, but does require a longer run time and may provide more ambiguous mappings. Finally, a reference genome and a file containing the corresponding protein sequences have to be provided.

To simplify the collection of all necessary data besides the PSMs, iPiG contains an optional automated file download tool. It provides a list of common organisms for which appropriate data is completely available. Currently, this includes *Caenorhabditis elegans*, *Danio rerio*, *Drosophila melanogaster*, *Gallus gallus*, *Homo sapiens*, *Mus musculus*, *Rattus norvegicus* and *Saccharomyces cerevisiae*. Data for further organisms have to be collected by hand. The data is downloaded from the ftp servers of the UCSC Genome Project and UniProt and can automatically be extracted after download.

Prior to the actual mapping, a gene quality control integrated within iPiG can be applied to establish the consistency of reference genes and amino acid translations. This is particularly crucial since the positioning of mappings to the reverse strand relies on the accordance of the coding sequence (cds) annotation with the length of the translated sequence.

The linking of peptides to genes can either be based on annotation-filtered sequence search (if available) or on sequence search only. Regarding the annotation, peptide identifications commonly contain a corresponding protein identifier (UniProtKB-ID, IPI and GI identifier are currently supported). Using the id mapping file, these identifiers can be linked to UniProtKB-AC, RefSeqs and Ensembl TRS identifiers, which are used as protein references in UCSC and Ensembl gene sets.

Once the linking of peptides to genes is available, the peptide sequence is searched against the corresponding amino acid translation of the gene to find the exact position within the protein. Multiple peptides can be linked to the same gene, thus the multiple-pattern search algorithm by Wu and Manber [16] is applied with peptides as patterns and a gene translation as text.

To map the position within a protein to the position within a gene, an array is filled with all coordinates of the cds of a gene in increasing order. The array corresponds to a potentially spliced transcript where splice sites are indicated by a difference of two adjacent coordinates greater than one. Using the position within the protein and the peptide length, the position in the gene can then be computed (see supplementary material). An illustrated example is given in Supplementary S1.

Commonly, several peptides cannot be linked to a gene using the reference or cannot be found within a linked gene. Thus, a second mapping pass is executed. This time, the multiple-pattern search algorithm is applied with all remaining peptides as patterns simultaneously to search in all gene translations for potential positions in proteins. All matches are then mapped to genome positions, as described above. This may result in several potential positions for a peptide and may not be as specific as the mapping based on references.

The output of iPiG consists of three file types: a browser extensible data (BED) file, a generic feature format version 3 (GFF3) file and a text file. The BED and the GFF3 formats are used to represent the peptide mappings as genomic features. They are most widely supported by common genome browsers such as the UCSC Genome Browser [3], Ensembl Genome Browser [4], GBrowse [5], IGB [6], IGV [7] or Geneious [8]. The peptides are grouped based on two properties, the uniqueness regarding the assignment of a PSM to one or more proteins and the score of a PSM. In the BED files, the uniqueness is denoted by different tracks and different scores are expressed by different color values. In the GFF3 files, uniqueness and score section groups are separated by different feature types containing the different properties as part of the feature type name. Further peptide identification properties are reported, such as the spectrum query, the peptide sequence, its charge and a shared count value indicating the number of mappings found for a specific peptide (each mapping is reported as a separate item). In addition to the mapping results, all unmapped peptides are reported separately.

## Results

**Figure 1:**
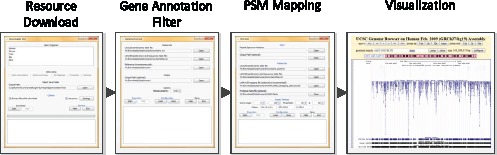
Overview of iPiG components and their graphical user interfaces. iPiG consists of the main program (PSM mapping) and two optional tools. In likely order of use, there is a download tool to retrieve necessary resource data easily and automatically, a gene filter to check consistency of required annotations and finally the mapping procedure itself. Although not part of iPiG, the output files allow the direct visualization in a genome browser as a final step.

iPiG is implemented in Java using the JDOM, Apache Xerces, Apache Commons Net and Apache Commons Compress libraries (all included) and is available as a stand alone, open-source software tool. As Java code, iPiG is platform independent and was tested on Windows, Linux and MacOS (requiring Java version 6). iPiG features a graphical user interface for the import of all five described input files and the setting of user-specific parameters. A visualization of the different graphical components of iPiG is shown in Figure 1.

**Figure 2:**
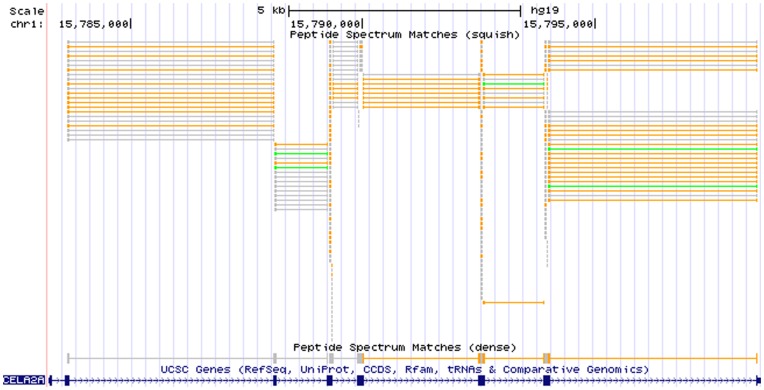
PSMs in a genome browser. Custom annotation tracks showing mapped peptides from an iPiG BED file imported into the UCSC Genome Browser. The peptide tracks only contain unique peptides that are mapped to referenced genes (annotation-filtered mapping). The peptides can be easily shown in context with other annotations such as the UCSC Genes (blue track). In particular, here PSMs are shown that mapped to the region of the gene CELA2A on chromosome one. The first PSM track (squish) shows all PSMs individually, where the different colors (grey, orange, green) code for different identification scores (low, mid, high). The second PSM track (dense) shows the PSMs overlapping and thus indicating a contiguous object. Both PSM tracks accurately represent the exon-intron structure of the corresponding gene, where thick blocks indicate exon parts and thin lines and arrows indicate intron parts resp. strand orientation.

We demonstrate the applicability of iPiG using a previously described data set [17]. Here, 32,125 peptide spectrum matches resulted from searching the acquired spectra from human cell lysates using Mascot against the human RefSeq database. iPiG was then applied to link the resulting identifications to the hg19 human reference genome provided by the UCSC Genome Browser. We used a standard desktop computer with an Intel Core 2 Duo 2.66 GHz CPU and 3 GB memory (actually limited to 1 GB by the Java virtual machine). The disk space consumption of all loaded resources (PSMs, human gene annotations, translations, id mapping file) was 140 MB (excluding data required for quality control). The matching of all 32,125 PSMs was completed in 129 seconds. In total, we obtained 20,591 mappings of PSMs using the annotation-filtered search and further 12,625 mappings using the sequence-only search. The resulting BED file was loaded into the UCSC Genome Browser for user-centric browsing. An example is shown in Figure 2.

Similar to gene expression analysis, the mapped peptides indicate where proteins are present and – if corresponding gene expression tracks are loaded – can validate gene expression hypotheses. The number of peptides mapped to one protein or gene may indicate an expression level. Increasing the zoom level will reveal mapping locations without overlaps as well as strand orientation and intron jumps. Additional information is either encoded by different colors (scores) or as part of the name of each feature (e.g. uniqueness, charge, orientation or the original peptide sequence itself). The example shows that the gene CELA2A on chromosome one is highly covered by various peptides of different length implying potential regions of interest. In particular, the higher quality peptides (orange and green bars) promise successful further investigation. The high number of higher quality peptides in the stacks of the squish view (first track) indicates the expression of CELA2A in a spectral counting manner. In addition, supported by the dense view (second track) the PSMs accurately represent the exon-intron structure of CELA2A. The visualization emphasizes the differing qualities of the initial peptides. In this example, no conclusion is solely based on lower confidence level peptide identifications (shown in gray), these do only support the findings of higher confidence identifications. However without higher confidence identifications available, these findings should be treated with more care.

iPiG takes advantage of the information gained in the protein identification process, especially with regard to the assignment of peptides to proteins. This allows a more specific and thereby faster peptide-gene mapping. Currently, the availability of mapping files is still limited. While iPiG can be run without these files the specificity of the results may be reduced and the run time is increased.

### Conclusion

The visualization of peptide and protein identifications in a genome browser bridges an important gap for proteogenomic data integration and visualization. Applications may range from protein expression visualization to transcript validation. It takes advantages of already available technology of genome browsers and allows easy data integration. It adheres to commonly used data formats and imports additional information from the peptide identification process to the genome browser visualization. iPiG is an easy-to-use and fast open-source tool that maps either specific to genes assigned by references or alternatively by sequence search alone.

## Supporting Information

Supplementary S1 **Supplementary material with graphical illustrations and descriptions of the iPiG mapping procedure and the use of the cdsmap.**
(PDF)

## References

[1] Castellana N, Bafna V (2010) Proteogenomics to discover the full coding content of genomes: A computational perspective. Journal of Proteomics 73: 2124–2135. 20620248 10.1016/j.jprot.2010.06.007 PMC2949459

[2] Renuse S, Chaerkady R, Pandey A (2011) Proteogenomics. PROTEOMICS 11: 620–630. 21246734 10.1002/pmic.201000615

[3] Kent WJ, Sugnet CW, Furey TS, Roskin KM, Pringle TH, et al (2002) The human genome browser at UCSC. Genome Research 12: 996–1006. 12045153 10.1101/gr.229102 PMC186604

[4] Hubbard T, Barker D, Birney E, Cameron G, Chen Y, et al (2002) The ensembl genome database project. Nucleic Acids Research 30: 38–41. 11752248 10.1093/nar/30.1.38 PMC99161

[5] Stein LD, Mungall C, Shu S, Caudy M, Mangone M, et al (2002) The generic genome browser: A building block for a model organism system database. Genome Research 12: 1599–1610. 12368253 10.1101/gr.403602 PMC187535

[6] Nicol JW, Helt GA, Blanchard SG, Raja A, Loraine AE (2009) The integrated genome browser: free software for distribution and exploration of genome-scale datasets. Bioinformatics 25: 2730–2731. 19654113 10.1093/bioinformatics/btp472 PMC2759552

[7] Robinson JT, Thorvaldsdóttir H, Winckler W, Guttman M, Lander ES, et al (2011) Integrative genomics viewer. Nature Biotechnology 29: 24–26. 10.1038/nbt.1754 PMC3346182 21221095

[8] Kearse M, Moir R, Wilson A, Stones-Havas S, Cheung M, et al (2012) Geneious basic: An in-tegrated and extendable desktop software platform for the organization and analysis of sequence data. Bioinformatics 28: 1647–1649. 22543367 10.1093/bioinformatics/bts199 PMC3371832

[9] Kalume D, Peri S, Reddy R, Zhong J, Okulate M, et al (2005) Genome annotation of anopheles gambiae using mass spectrometry-derived data. BMC Genomics 6: 128. 16171517 10.1186/1471-2164-6-128 PMC1249570

[10] Dowell R, Jokerst R, Day A, Eddy S, Stein L (2001) The distributed annotation system. BMC Bioinformatics 2: 7. 11667947 10.1186/1471-2105-2-7 PMC58584

[11] Sanders W, Wang N, Bridges S, Malone B, Dandass Y, et al (2011) The proteogenomic mapping tool. BMC Bioinformatics 12: 115. 21513508 10.1186/1471-2105-12-115 PMC3107813

[12] Deutsch EW (2012) File formats commonly used in mass spectrometry proteomics. Molecular & Cellular Proteomics doi:10.1074/mcp.R112.019695. 10.1074/mcp.R112.019695 PMC3518119 22956731

[13] Kessner D, Chambers M, Burke R, Agus D, Mallick P (2008) ProteoWizard: open source software for rapid proteomics tools development. Bioinformatics 24: 2534–2536. 18606607 10.1093/bioinformatics/btn323 PMC2732273

[14] Bradshaw RA, Burlingame AL, Carr S, Aebersold R (2006) Reporting protein identification data – the next generation of guidelines. Molecular & Cellular Proteomics 5: 787–788. 16670253 10.1074/mcp.E600005-MCP200

[15] Renard BY, Timm W, Kirchner M, Steen JAJ, Hamprecht FA, et al (2010) Estimating the confidence of peptide identifications without decoy databases. Analytical Chemistry 82: 4314–4318. 20455556 10.1021/ac902892j

[16] Wu S, Manber U (1994) A fast algorithm for multi-pattern searching. Technical Report TR94–17, Department of Computer Science, The University of Arizona.

[17] Renard BY, Kirchner M, Monigatti F, Ivanov AR, Rappsilber J, et al (2009) When less can yield more – computational preprocessing of MS/MS spectra for peptide identification. PROTEOMICS 9: 4978–4984. 19743429 10.1002/pmic.200900326

