# Supplementary material for: iPiG: Integrating Peptide Spectrum Matches into Genome Browser Visualizations
Source: PLoS One. 2012 Dec 4;7(12):e50246. doi: 10.1371/journal.pone.0050246 (PMC3514238; doi:10.1371/journal.pone.0050246)
Supplement: Supplementary S1 — Supplementary material with graphical illustrations and descriptions of the iPiG mapping procedure and the use of the cdsmap. (PDF) [file pone.0050246.s001.pdf]

Supplementary Material to iPiG: Integrating Peptide Spectrum Matches Into Genome Browser Visualizations

Mathias Kuhring and Bernhard Y. Renard[1]
Research Group Bioinformatics (NG4), Robert Koch-Institute, Berlin, Germany

iPiG Workflow Illustration

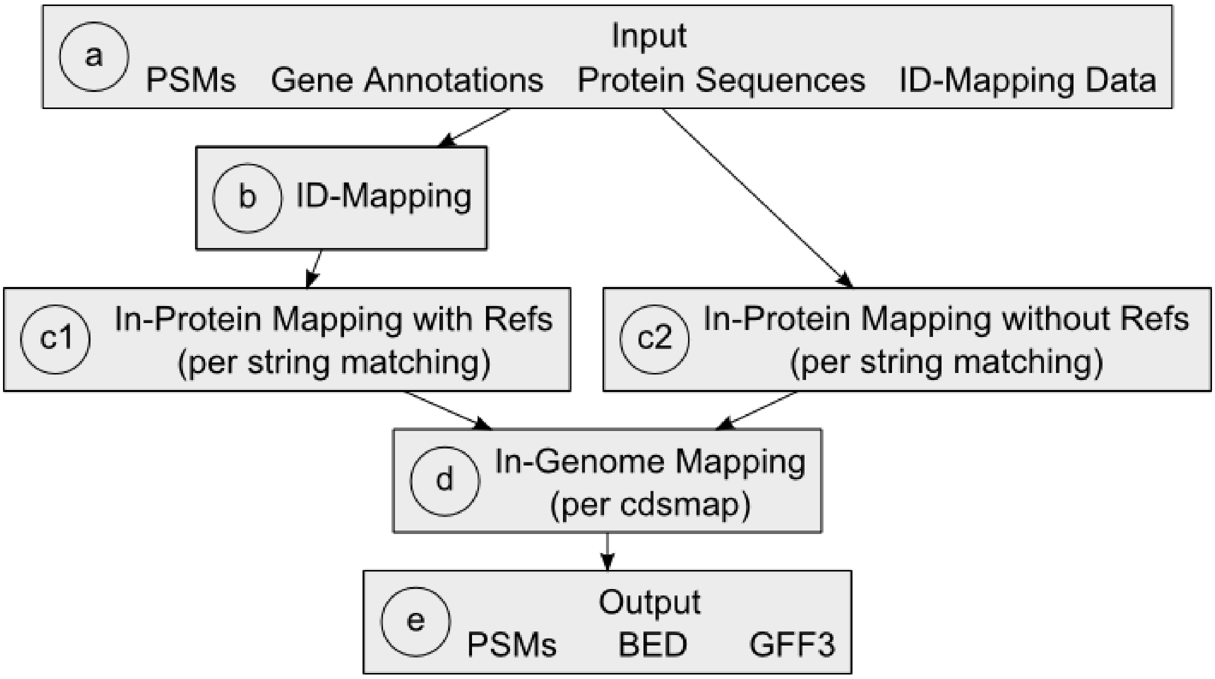

Figure S1: Flowchart describing the iPiG workflow. Peptide spectrum matches (PSMs) of interest and the required resources for the mapping procedure are imported (a). If the protein identifier of a PSM can be linked to a gene (b), the peptide is mapped to the corresponding amino acid translation of the gene (c1). Otherwise, the peptide is mapped to all matching gene translations (c2). The position in the protein is converted into the position in the genome using a cdsmap (d). Finally, the located PSMs are provided as tracks for genome browsers in standard formats such as BED and GFF3.

Cdsmap Example

The cdsmap is used to map the position within a protein to a position in the corresponding gene and thus the genome. An array (called cdsmap) is filled with all positions/coordinates of the coding sequence (cds) of a gene in increasing order. It is built from gene annotations on exon starts and ends.

A cdsmap represents a potentially spliced transcript and nucleotides are represented by their chromosome positions (in increasing order). It has three times the length of the corresponding protein, since three nucleotide positions correspond to one codon which codes for one amino

1 Contact: RenardB@rki.de

acid in the protein. Splice sites are indicated by a difference of two adjacent coordinates greater than one.

The peptide position within the protein and the peptide length can be used to obtain the subset of the array coding for the peptide. Finally, taking the first and the last element from the subset yields the genome position boundaries of the peptide, whereas exon spanning peptides are determined by the adjacent coordinates with differences greater than one.

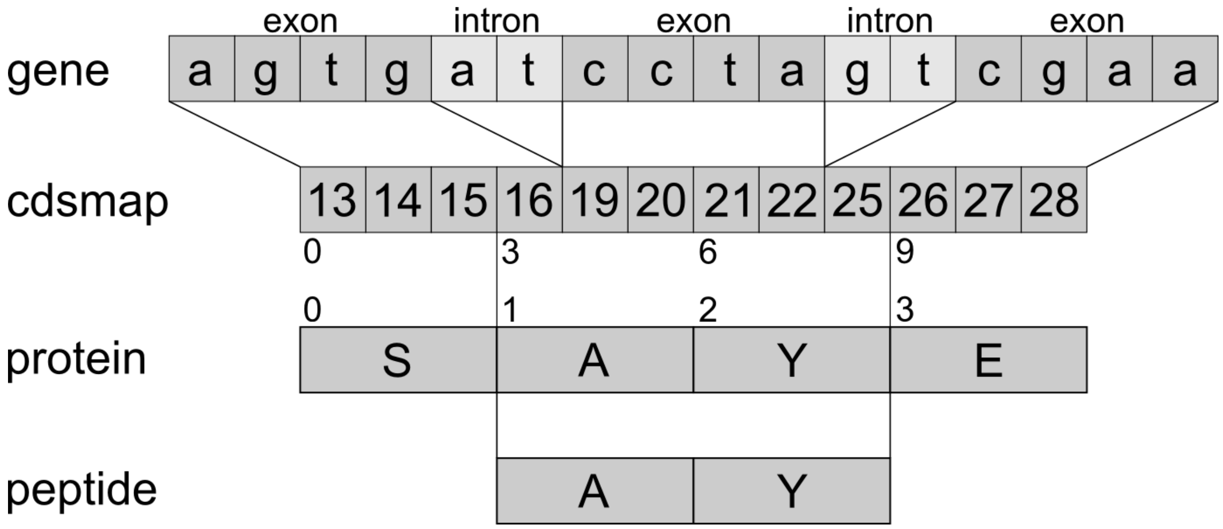

Figure S2: Schematic example illustrating the usage of the cdsmap for a gene starting at position 13 and ending at position 28 on a chromosome. Given a peptide that matched to the corresponding protein (by string matching), the match positions multiplied by three can be used to extract a cdsmap subpart that now corresponds to the transcript coding the peptide. The exact genomic position can then be extracted.
